# Heterogeneity of Small Intestinal Neuroendocrine Tumors Metastasis: Biologic Patterns of a Series with Virchow’s Node Involvement

**DOI:** 10.3390/cancers14040913

**Published:** 2022-02-12

**Authors:** Maria Wedin, Marina Tsoli, Göran Wallin, Eva Tiensuu Janson, Anna Koumarianou, Gregory Kaltsas, Kosmas Daskalakis

**Affiliations:** 1 Department of Surgery, Faculty of Medicine and Health, Örebro University, 70185 Örebro, Sweden; maria.wedin@regionorebrolan.se (M.W.); goran.wallin@regionorebrolan.se (G.W.); 2 1st Department of Propaedeutic Internal Medicine, National and Kapodistrian, University of Athens, 11527 Athens, Greece; martso.mt@gmail.com (M.T.); gkaltsas@med.uoa.gr (G.K.); 3 Department of Medical Sciences, Uppsala University, 75185 Uppsala, Sweden; eva.tiensuu_janson@medsci.uu.se; 4 Hematology-Oncology Unit, Fourth Department of Internal Medicine, Attikon Hospital, Medical School, National and Kapodistrian University of Athens, 12462 Athens, Greece; akoumari@yahoo.com; 5 2nd Department of Surgery, “Korgialenio-Benakio”, Red Cross General Hospital, 11526 Athens, Greece

**Keywords:** small intestinal neuroendocrine neoplasm, Virchow’s node metastasis

## Abstract

**Simple Summary:**

Virchow’s node metastasis (VM) refers to the involvement of the left supraclavicular lymph nodes at the junction of the thoracic duct and the left subclavian vein. Generally, VM is considered by clinicians to be a strong indicator of metastatic abdominal malignancy, and its dismal prognostic significance has previously been described in patients with metastatic gastric and ovarian cancer. To date, comprehensive descriptions of patients with small intestinal neuroendocrine tumors (SI-NETs) and rare metastatic manifestations, including that of VM, are sparse. In the present study from two tertiary referral centers, the prevalence of the VM secondary to SI-NET primaries was found to be 3.9%. VM was more often encountered in patients with higher-grade tumors and established disseminated disease to distant para-aortic lymph nodes. However, the presence of VM did not yield any negative prognostic impact in patient outcomes when compared to age- and sex-matched patients of similar grade with distant metastases confined in the abdomen

**Abstract:**

Small intestinal neuroendocrine tumors (SI-NETs) may rarely metastasize to the left supraclavicular lymph nodes, also known as Virchow’s node metastasis (VM). Data on prevalence, prognostic significance, and clinical course of disease for SI-NET patients with VM is limited. In this retrospective analysis of 230 SI-NET patients treated at two tertiary referral centers, we found nine patients with VM (prevalence 3.9%). Among those, there were 5 females and median age at SI-NET and VM diagnosis was 61 and 65 years, respectively. Two patients had G1 tumors and five G2, while two tumors were of unspecified grade (median Ki67: 7%, range 2–15%). Four patients presented with synchronous VM, whereas five developed metachronous VM after a median of twenty-four months (range: 4.8–117.6 months). Hepatic metastases were present in seven patients, extrahepatic metastases (EM) in eight (six para-aortic distant lymph node metastases, one lung and one pancreatic metastasis), whereas peritoneal carcinomatosis (PC) in two patients. We used a control group of 18 age- and sex-matched SI-NET patients from the same cohort with stage IV disease but no extra-abdominal metastases. There was no difference in best-recorded response to first line treatment according to RECIST 1.1 as well as progression-free survival (PFS) between patients with VM and those in the control group (Chi-square test *p* = 0.516; PFS 71.7 vs. 106.9 months [95% CI 38.1–175.8]; log-rank *p* = 0.855). In addition, median overall survival (OS) of SI-NET patients with VM did not differ from those in the control group (138.6 [95% CI 17.2–260] vs. 109.9 [95% CI 91.7–128] months; log-rank *p* = 0.533). In conclusion, VM, although relatively rare in patients with SI-NETs, is more often encountered in patients with G2 tumors and established distant para-aortic lymph node metastases. The presence of VM in SI-NET patients does not seem to impact patients’ survival outcomes and treatment responses, when compared to age- and sex-matched SI-NET patients with stage IV disease confined in the abdomen.

## 1. Introduction

Small intestinal neuroendocrine tumors (SI-NETs) have an indolent clinical course and are often diagnosed at a late stage. A high proportion of patients (approximately up to 60%) are diagnosed with stage IV tumors (i.e., distant metastases), most commonly to the liver [1,2]. Distant abdominal lymph node metastases in the root of the mesentery and/or in the retroperitoneal space and para-aortic region occur in up to 18% of SI-NET patients at presentation and are often present in cases with locally advanced disease commonly associated with extensive mesenteric fibrosis [2,3,4]. However, extra-abdominal distant metastases, mainly to the bones and the lungs, are encountered in only up to 6.1% of SI-NET patients at presentation. Importantly, distant extrahepatic metastases to the para-aortic lymph nodes, bones, and lungs have been recognized as independent prognostic factors for survival [2,5,6].

Virchow’s node metastasis (VM) refers to involvement of the left supraclavicular lymph nodes at the junction of the thoracic duct and the left subclavian vein. Generally, VM is considered by clinicians to be a strong indicator of metastatic abdominal malignancy [7]. Commonly, it is caused by metastatic gastric adenocarcinoma, but can also be seen in other gastrointestinal, pelvic, and thoracic malignancies (which metastasize to the left supraclavicular lymph nodes by means of migration of tumor emboli through the thoracic duct). Notably, abdominal, and pelvic tumors uniformly metastasize to the lymph nodes of the left supraclavicular fossa, whereas malignancies of the head and neck, thorax, breast, and skin (as well as lymphomas) exhibit no significant difference in laterality [8]. The prognostic significance of VM has previously been described in patients with metastatic gastric and ovarian cancer [9,10].

The prevalence of VM in the subset of SI-NETs is largely unknown, and currently we have very limited data regarding the clinical course and management of SI-NET patients with this manifestation. In this study conducted on patients from two European tertiary referral centers, we aimed to identify the prevalence of VM among SI-NET patients and its impact in patient outcomes, also shedding light on clinico-pathological features and diagnostics.

## 2. Material and Methods

Data were extracted retrospectively from the medical records of 230 consecutive SI-NET patients admitted to two tertiary referral centers: the Örebro University Hospital, Örebro, Sweden and the EKPA-Laiko Hospital, Athens, Greece. Only patients with a definite histopathological SI-NET diagnosis, well-differentiated neoplasms and clinically or radiologically confirmed VM were included. Patients with neuroendocrine carcinomas were not included. The SI-NET diagnoses were made between the 20th August 1998 and the 8th March 2021; and patients were followed until death or the 1st April 2021. Demographics (age and gender) as well as disease characteristics (the timing of VM diagnosis in relation to SI-NET diagnosis, octreoscan/gallium (Ga)-68 positron emission tomography [PET] positivity and clinico-pathological parameters) were registered. The presence of VM was recorded either at SI-NET diagnosis or subsequently during the disease follow-up. An age- and sex-matched control group with stage IV SI-NET without extra-abdominal metastases from the two participating centers was used for comparison. Patients with VM and those in the control group were diagnosed within the same time frame between 2004 and 2020 in order to eliminate confounders related to evolving diagnostic tools and changing treatment algorithms. To ensure the quality of data reporting, we followed the STROBE statement [11].

### 2.1. Ethics Statement

The study was conducted according to the 1975 Declaration of Helsinki and approved by the pertinent ethics review boards at the participating centers. Written informed consent was obtained from all of the study participants.

### 2.2. Diagnosis of and Disease Classification of SI-NET

SI-NET diagnosis was made on the basis of histopathological confirmation, in combination with serum and urine biomarkers (chromogranin-A [CgA] and 5-hydroxy-indoleacetic acid [5-HIAA]) as well as cross-sectional and functional imaging. VM diagnosis secondary to SI-NET was confirmed on somatostatin receptor imaging and/or histopathological examination of surgical/biopsy material.

Each center followed the ENETS guidelines for surveillance protocols with sequential imaging and serum and urine biomarkers. At diagnosis, patients underwent cross-sectional imaging with either CT scan or magnetic resonance imaging (MRI) of the abdomen and CT of the thorax. Subsequently, cross-sectional imaging with CT or MRI scan of the abdomen was regularly performed to assess the tumor load, and to evaluate treatment effects and the recurrence or progression of disease [12]. Functional imaging with somatostatin receptor scintigraphy and/or PET using ^68^Ga-DOTATOC as tracer was performed at diagnosis and when indicated during follow-up as per ENETS guidelines [12]. Morphological imaging of the abdomen (CT or MRI) was reviewed and the highest liver tumor load (LTL) was recorded. The following staging system was used to describe the stage of liver involvement: stage 1, fewer than 5 metastases confined in 1 lobe; stage 2, bi-lobar and/or 5 to 10 metastases; and stage 3, more than 10 metastases or diffuse metastatic disease. Tumor grade was determined from primary and lymph node specimens and/or liver biopsies according to the Ki-67 proliferation index. We used the 2019 WHO classification systems for grading SI-NETs [13]. For staging, we used the 8th edition of the American Joint Committee on Cancer (AJCC) [14]. The Charlson comorbidity index (CCI) score was estimated for each patient in this study [15].

### 2.3. Statistics

All statistical analyses (frequencies, descriptive statistics, χ^2,^ Kaplan-Meier curves, log-rank tests, and Cox-regression analysis) were carried out with the SPSS v23.0 software package (IBM SPSS Statistics, Armonk, NY, USA). Progression-free (PFS) and overall survival (OS) were analyzed using the Kaplan-Meier method. To calculate the PFS and OS of patients with VM, the onset or first diagnosis of VM was used as the baseline in order to eliminate immortal time bias. Accordingly, for the comparison with the stage IV patients without VM, onset of stage IV was set as the baseline for survival estimates. Pearson chi-square test for best recorded response to first line treatment and log-rank test (Mantel-Cox) analysis to assess differences in PFS and OS estimates were used. Tests were two-sided, *p* < 0.05 was considered statistically significant, and the 95% confidence interval (CI) was given for survival estimates.

## 3. Results

### 3.1. Clinical Features, Patient and Tumor Characteristics in the VM Group

Nine patients with VM were identified among 230 SI-NET patients in the databases of the two participating centers. All VM identified developed from well-differentiated SI-NET. The median age was 61 years at initial SI-NET diagnosis (range: 38–76.2) and 65 years at VM diagnosis (range: 38–76.3). The female-to-male ratio was 1.25 (5/4). Four patients had synchronous VM (i.e., identified at the time of initial SI-NET diagnosis), whilst five patients had metachronous VM diagnosed during follow-up at a median time of 24 months from SI-NET diagnosis (range: 4.8–117.6 months). All patients with VM had well-differentiated SI-NETs. Two patients had G1 tumors, five G2, and two cases had no Ki-67 index or mitosis counts for grading (median Ki67: 7%, range 2–15%).

Two patients manifested carcinoid syndrome at the time of VM diagnosis. We did not encounter any cases with thoracic outlet syndrome, phrenic neuropathy or Horner syndrome.

Serum levels of chromogranin A and urine or serum levels of 5HIAA were measured and were elevated in seven and six patients with VM, respectively.

The CCI score was ≥4 in four patients. The median follow-up time was 67.2 months (range: 8.6–147.7) after VM diagnosis.

### 3.2. Concomitant Metastatic Lesions

All VM co-existed with other concomitant distant metastatic lesions (Table 1). The majority of patients had liver metastases (*n* = 8) and para-aortic lymph node metastases (*n* = 6), one had a pancreatic metastasis and one had lung metastases. Five patients exhibited advanced mesenteric fibrosis, and two had peritoneal carcinomatosis. Among patients with liver metastases and VM, five had a more advanced liver tumor burden of stage 2 (*n* = 2) and 3 (*n* = 3, Table 1). There were no evident bone metastases in the study group.

### 3.3. VM Detection and Diagnostic Approaches

**Figure 1:**
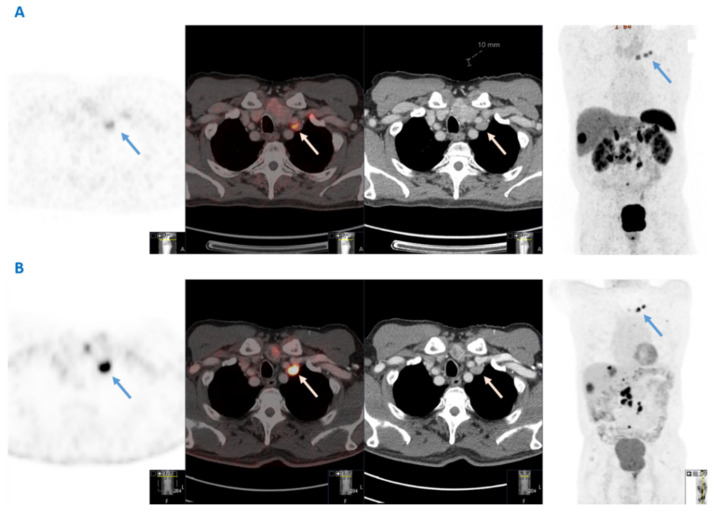
Dual functional imaging of a patient with a G2 small intestinal neuroendocrine neoplasm and Virchow’s node metastases (blue and white arrows), both ^68^Ga-DOTATATE- and ^18^F-FDG avid. (**A**) From left to right: ^68^Ga-DOTATATE PET; fusion; computed tomography; and maximum intensity projection (MIP) images (**B**) From left to right: ^18^F-FDG PET; fusion; computed tomography; and maximum intensity projection (MIP) images.

The initial detection of VM in the nine patients identified in this study was made by clinical examination in two patients, conventional CT in two patients and ^68^Ga-DOTATATE PET-CT in five patients. Overall, in two out of four patients with synchronous VM, the presence of VM itself led to the SI-NET diagnosis. Importantly, all VM lesions were somatostatin receptor positive in this series in subsequent functional imaging. Histopathological confirmation of VM secondary to SI-NET was obtained in five patients for diagnostic purposes through core needle biopsy (*n* = 3) or surgical resection (*n* = 2) of the left supraclavicular nodes. ^18^F-FDG PET-CT was performed in only three patients, in two of whom the VM lesion was FDG positive. The CT scan, ^68^Ga-DOTATATE PET-CT and ^18^F-FDG PET-CT imaging of a SI-NET patient with VM are shown in Figure 1.

### 3.4. Surgical and Systemic Treatments

Variable treatments were administered throughout the prolonged disease course. Seven patients had surgical resection of the primary tumor. At VM diagnosis, eight patients were treated with somatostatin analogs (SSAs) and one patient received molecular targeted therapy (MTT) with everolimus.

### 3.5. Baseline Characteristics and Comparisons with the Age- and Sex-Matched Control Group

Eighteen age- and sex-matched individuals (2:1 matching) with stage IV disease and no EM were identified. All of the patients in the control group had well-differentiated tumors. The median age was 65.7 years at SI-NET diagnosis (range: 38.9–75.9). Eight patients had G1 tumors and ten G2 tumors (median Ki67: 4%, range 1–20%). Notably, in the initial cohort of 230 SINEN patients, 132 patients had G1 tumors, 76 G2, 3 G3 and 15 of unspecified grade (chi-square *p* = 0.049 for grade 1 and 2 between patients with and without VM in the whole cohort).

All of the patients in the control group had liver metastases. Four patients had intra-abdominal para-aortic lymph node metastases and three had peritoneal carcinomatosis. With regards to liver tumor burden, eleven had a more advanced extent of liver involvement (stage 2, *n* = 4; and stage 3, *n* = 7, Table 1). Ten patients manifested carcinoid syndrome at the time of diagnosis.

Serum levels of chromogranin A and serum or urine 5HIAA were measured and were elevated in four and six patients, respectively. However, in many cases baseline biomarker concentrations were not available owing to missing data. The CCI score was ≥4 in 10 patients. Baseline comparisons on patient and tumor characteristics between patients with VM and patients in the control group are presented in Table 1. In particular, comparative analysis between the baseline characteristics of both groups only revealed a significant difference in the presence of distant para-aortic lymph node metastases, that were more often encountered in patients with VM (*p* = 0.039). Other parameters, including grade (*p* = 0.529), liver tumor load (*p* = 0.375), peritoneal carcinomatosis (*p* = 0.999), and biomarker levels (*p* = 0.999 for CgA, and *p* = 0.619 for 5 = HIAA) did not yield any significant difference between patients with VM and controls, implying that the two groups were well-balanced (Table 1). Finally, the first line treatments administered to patients in the control group did not differ from those given to patients with VM (*p* = 0.759, Table 1).

### 3.6. Clinical Relevance of VM

To study the clinical relevance of VM, we compared best recorded responses to first line treatment and assessed VM prognostic value comparing the median PFS and OS of patients with and without VM. The five-year OS rate for patients with VM was estimated at 80%. There was no difference in best-recorded response to 1st line treatment according to RECIST 1.1 as well as PFS between patients with VM and those in the control group (Chi-square test *p* = 0.516; PFS 71.7 vs. 106.9 months [95% CI 38.1–175.8]; log-rank *p* = 0.855; Supplementary Figure S1). In addition, median OS of SI-NET patients with VM did not differ from those in the control group (138.6 [95% CI 17.2–260] vs. 109.9 [95% CI 91.7–128] months; log-rank *p* = 0.533; Supplementary Figure S2).

## 4. Discussion

In our study, the prevalence of the VM secondary to SI-NET primaries was found to be 3.9% (2.2% for metachronous cases). VM was more often encountered in patients with G2 tumors and established stage IV disease with already established extrahepatic metastases, mainly to distant para-aortic lymph nodes. However, its presence was not found to be a prognostic factor for PFS and/or OS when compared to age- and sex- matched stage IV SI-NET patients. To date, comprehensive descriptions and survival analysis of SI-NET patients with rare metastatic manifestations, including that of VM, are sparse [5,6,16,17,18]. The prevalence of VM among patients with other abdominal and thoracic malignancies has previously been estimated up to 2.8%. Among those patients who presented with VM involvement, the highest frequency was in patients with lung cancer followed by cancer of the pancreas, esophagus, kidney, ovary, testicle, stomach, prostate, uterus, and rectum [19].

All patients with VM secondary to SI-NET in this series were related to well-differentiated tumors with advanced stage IV disease. The diagnosis of VM was synchronous in four patients and metachronous in five patients. In two patients with synchronous VM, the presence of VM itself led to the SI-NET diagnosis. Importantly, VM was confirmed in octreoscan or DOTATATE PET-CT in all of the patients in this series, as all VMs originated from well-differentiated tumors. Due to the increased availability of new molecular imaging modalities, in particular ^68^Ga-DOTATATE PET-CT, the diagnosis of rare metastatic manifestations of NETs is indeed steadily increasing [16,20]. As the prevalence of VM is relatively low and these lesions were clearly recognized in functional imaging with ^68^Ga-DOTATATE PET-CT, which is currently applied both at diagnosis and during disease surveillance, we could advocate in favor of routine screening for VM with ^68^Ga-DOTATATE PET-CT at baseline in SI-NET patients. However, no clear association between the presence of VM and unfavorable prognosis was evident in our series.

In addition, G1 and G2 SI-NET patients may also have ^18^F-FDG-positive tumors initially or may develop ^18^F-FDG-positive lesions during follow-up, as demonstrated in Figure 1, with important implications for therapy optimization and disease surveillance. Dual functional imaging could probably be considered prior to treatment initiation to delineate tumor somatostatin receptor expression and glycolytic metabolic activity in the context of a personalized treatment strategy at least in G2 patients with Ki67 in the higher levels. Baseline dual-functional imaging assessment in higher grade SI-NETs with advanced tumor burden could be used for the selection of patients requiring PRRT or other systemic treatments as well as the prognostic evaluation of the disease [21]. Therefore, as G2 tumors were more often associated with the presence of VM, dual functional imaging could probably be considered in a subset of SINET patients of higher grade and more advanced disease at baseline, with the aims to accurately define metastatic extent and tumor aggressiveness.

Considering its anatomy, VM could result in certain complications secondary to mass effect [22]. Neurogenic and/or vascular thoracic outlet syndrome, have been reported in the literature [23], as well as unilateral phrenic neuropathy with ipsilateral diaphragmatic weakness and Horner syndrome [24]. However, we did not encounter the aforementioned symptomatology in the present series of VM secondary to SI-NET. Extensive fibrotic reactions in the root of the mesentery along with para-aortic lymph node metastases were often present in the VM positive SI-NET patients of our study cohort. Presumably, tumor emboli may accompany central mesenteric lymph node metastases and associated fibrosis with retroperitoneal extension in the setting of locally advanced SI-NETs. In these cases, neoplastic emboli may bypass the portal circulation and drain directly into the systemic circulation through retroperitoneal lymphatic spread and the thoracic duct resulting not only in VM, but also in other extra-abdominal metastatic manifestations, present in the study cohort [3].

Survival in patients with VM is generally poor in non-neuroendocrine malignancies, such as cases with gastric or ovarian cancer [9,10]. Traditionally, VM appeared as a terminal manifestation of generalized cancer. However, possibly due to the indolent nature of SI-NET, we could not confirm an increased mortality risk in our series. Indeed, comparable PFS and OS rates were evident between patients with and without VM. This could also be due to overrepresentation of SI-NET patients with G2 tumors and advanced-stage IV disease in our control group from the two tertiary referral centers.

With regards to surgical treatment, contemporary evidence does not provide unambiguous support for the use of debulking procedures nor metastasectomies [25]. Nevertheless, the metastatic pattern of para-aortic lymph node metastases and the presence of VM itself in the setting of disseminated SI-NETs, changes the nature of the cancer to a systemic disease; as such, surgery is generally not considered for this lesion apart from diagnostic purposes. Generally, once VM has been identified, the issue of systemic NEN treatments becomes pertinent. No standard treatment modality has been established for distant extrahepatic metastases, including para-aortic lymph nodes and VM. To date, the systemic treatment choice depends mainly on tumor histolopathology, metastatic extent, functionality and patient’s performance status, as per latest ENETS and ESMO guidelines [26,27]. SSA, PRRT, and in selected cases the mTOR inhibitor everolimus, remain the mainstay of systemic treatment for SI-NET patients with advanced stage IV disease, including the VM cases of our study [27,28].

There are several limitations to our study. This was a retrospective analysis, and there were only nine SI-NET patients with VM. Due to the small sample size of the study and the lack of statistical power, combined with the relatively favorable survival outcomes of SI-NET patients, PFS and OS analyses findings should be interpreted with caution. Ideally, a larger sample size and a comparative control group with stage IV patients and distant para-aortic lymph nodes in the mesenteric root, the retroperitoneum and/or the pelvis, but still confined in the abdomen, could be used to better delineate the prognostic implications of VM in SINETs. However, due to the relative scarcity of these manifestations, we were not able to identify an adequate number of such patients in the initial cohort to proceed with meaningful statistical analysis. In addition, treatment algorithms and imaging modalities have changed significantly over the study period, also implying that some cases with VM might have been initially missed with CT or octreoscan. Indeed, SI-NET heterogeneity, treatment factors, such as the quality of surgery in different centers, differences in diagnostic approaches and systemic treatments, may all have confounded our results. This study is also limited by a referral bias to the two tertiary centers involved, as well a lack of central pathology review, although an expert pathologist was engaged in each collaborating center. We conducted statistical analysis of patients’ outcomes using an age- and sex-matched control group of stage IV SI-NET patients to reduce confounding. The strength of this study is that it provides an epidemiologic picture of SI-NET associated with a rare metastatic manifestation to the left supraclavicular lymph nodes with a prevalence and prognosis assessment.

## 5. Conclusions

In conclusion, this is the first comprehensive analysis of SI-NET patients with VM, which demonstrates a relatively low prevalence of VM among patients with SI-NETs. In particular, we reviewed the medical files of 230 patients from two tertiary referral centers, and we report that the occurrence of VM enlargement is 3.9%. VM was more often encountered in patients with G2 tumors and was commonly detected by functional imaging with ^68^Ga-DOTATATE PET-CT. Concomitant metastases to para-aortic lymph nodes were also more often present. Unlikely to other primaries, enlarged VM in SINET is not ominous and does not seem to be associated with worse survival. Our study indicates that although it is not a frequently occurring event, enlarged VM may occur in well-differentiated SINETs. In addition, its recognition does not necessarily imply a second different primary, as it is commonly substantiated through functional imaging and histopathology. Therefore, NET physicians should be aware of this metastatic occurrence and its lack of prognostic significance for patient survival in SINETs. Patients should be treated based on the metastatic nature of VM with systemic therapy. Although local treatment with radiotherapy can be allegedly applied in similar cases of bulky symptomatic nodal enlargement or refractory disease, we have no evidence supporting its upfront use in SINETs nor the use of specific local therapies and PRRTs in this setting to generate some sort of treatment algorithm.

Further international collaborations and studies addressing the clinical impact of rare metastatic manifestations in NENs are needed to increase clinicians’ awareness and elucidate further aspects of SI-NET diagnostics and management, contributing to a more individualized therapeutic strategy as clinically indicated per patient.

## Figures and Tables

**Table 1:** Patients’ baseline characteristics at the time of Virchow’s metastasis (VM) diagnosis (*n* = 9) and characteristics of the age- and sex-matched control group with stage IV disease (*n* = 18).

Characteristics	VM Group (*n* = 9)	Control Group (*n* = 18)	*p*-Value ^†^
Gender			N/A
Female	5	10	
Male	4	8	
Median age, years (range)			N/A
SINET diagnosis	60.9 (36.1–76.2)	65.74 (38.9–75.2)	
VM diagnosis	64.3 (36.1–76.3)	N/A	
WHO classification			0.529
G1	2	8	
G2	5	10	
Unknown	2	N/A	
Primary tumor multifocality			
No	5	10	0.999
Yes	2	5	
Unknown	2	3	
Primary tumor size (mm)			0.887
Median (range)	22 (7–60)	18 (10–53)	
Mesenteric fibrosis			0.219
No	4	13	
Yes	5	5	
Distant para-aortic lymph nodes	6	4	0.039
Concomitant distant metastases			
Liver	7	18	0.103
Lung	1	0	0.333
Bone	0	0	N/A
Other (pancreatic)	1	0	0.333
Liver tumor load			0.375
0	1	0	
1	0	2	
2	2	4	
3	3	7	
Unknown	3	5	
Peritoneal carcinomatosis			0.999
No	7	15	
Yes	2	3	
Octreoscan/^68^ Ga positivity			
No	0	1	0.999
Yes	9	17	
Chromogranin A			0.999
Normal	2	6	
Elevated	7	4	
Unknown	0	8	
5-HIAA			0.619
Normal	3	4	
Elevated	6	6	
Unknown	0	7	
Carcinoid syndrome			0.217
No	6	8	
Yes	3	10	
Prior resection of SI-NET primary			
No	2	5	0.999
Yes	7	13	
Systemic 1st line treatment at baseline			0.759
SSA	8	13	
IF-a	0	0	
PRRT	0	1	
MTT	1	2	
Chemotherapy	0	1	
Charlson Comorbidity Index			0.622
0	0	0	
1	2	3	
2	2	2	
3	1	3	
≥4	4	10	

Abbreviations: 5-HIAA, 5-hydroxyindoloaceatic acid; IF-a, interferon-alpha; MTT, molecular targeted therapy; N/A, non applicable; PRRT, Peptide receptor radionuclide therapy; SINET, small intestinal neuroendocrine tumor; SSA, somatostatin analog. ^†^ *p*-values were computed with the Pearson’s chi-square test, the Fisher’s exact test or the Mann-Whitney test, as appropriate.

## Data Availability

Individual patient data included in the present study are available upon request from the corresponding author.

## Supplementary Materials

The following supporting information can be downloaded at: https://www.mdpi.com/article/10.3390/cancers14040913/s1. Figure S1. Kaplan–Meier progression-free survival analysis to 1st line treatment of patients with small intestinal neuroendocrine tumors and Virchow’s node metastasis and those matched for age and gender but without extrahepatic metastases. *p* = 0.855 (log rank test); Figure S2. Kaplan–Meier overall survival analysis of patients with small intestinal neuroendocrine tumors and Virchow’s node metastasis and those matched for age and gender but without extrahepatic metastases. *p* = 0.533 (log rank test).
