# Supplementary material for: Heterogeneity of Small Intestinal Neuroendocrine Tumors Metastasis: Biologic Patterns of a Series with Virchow’s Node Involvement
Source: Cancers (Basel). 2022 Feb 12;14(4):913. doi: 10.3390/cancers14040913 (PMC8869999; doi:10.3390/cancers14040913)
Supplement: Supplementary file 1 [file cancers-14-00913-s001.zip › cancers-1549798-supplementary.pdf]

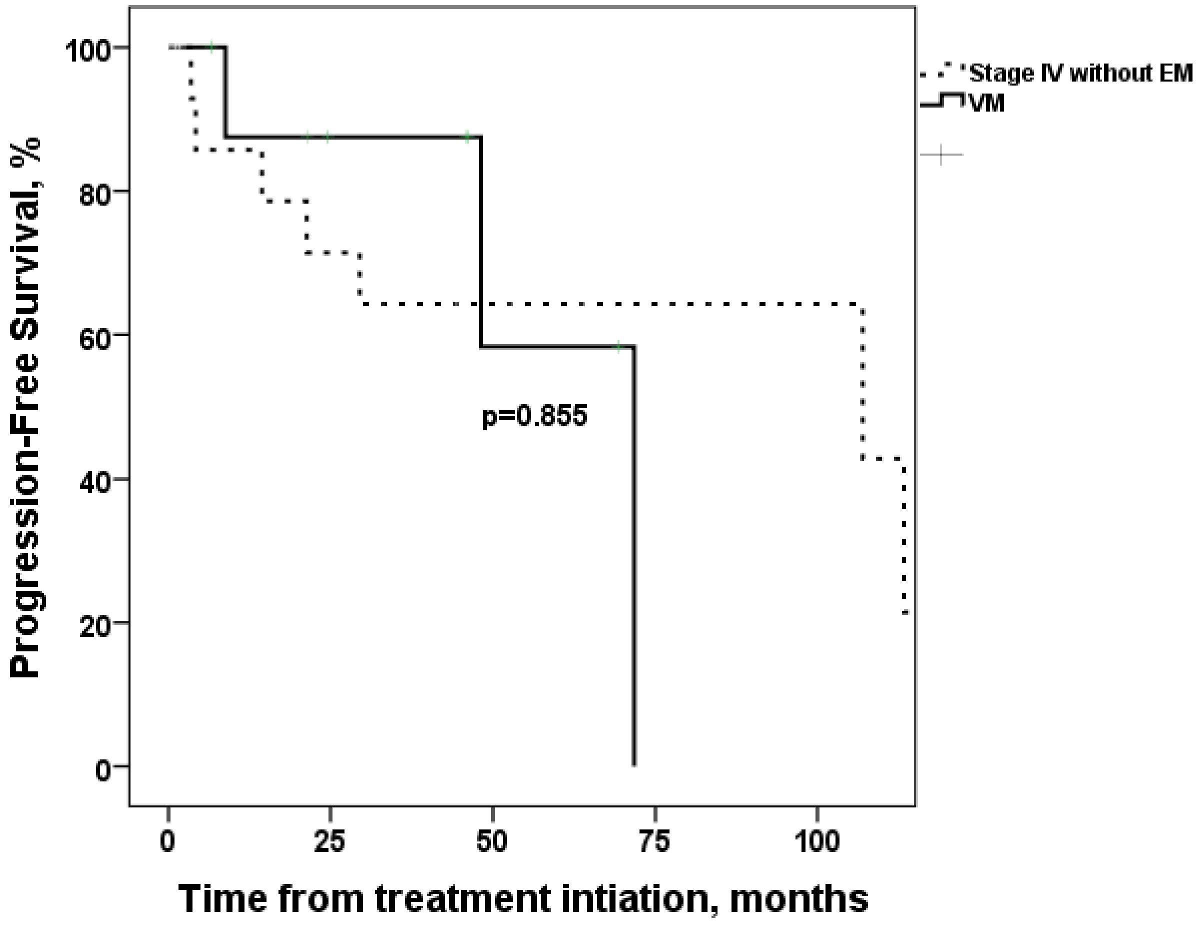

No. patients at risk.

**Virchow's node metastasis**	**9**	**5**	**2**	**1**
**Stage IV without extrahepatic metastases**	**18**	**8**	**5**	**2**

Supplementary Figure S1. Kaplan–Meier progression-free survival analysis to 1st line treatment of patients with small intestinal neuroendocrine tumors and Virchow's node metastasis and those matched for age and gender but without extrahepatic metastases. *P* = 0.855 (log rank test).

Abbreviations: EM, extra-abdominal metastases; VM, Virchow's metastasis

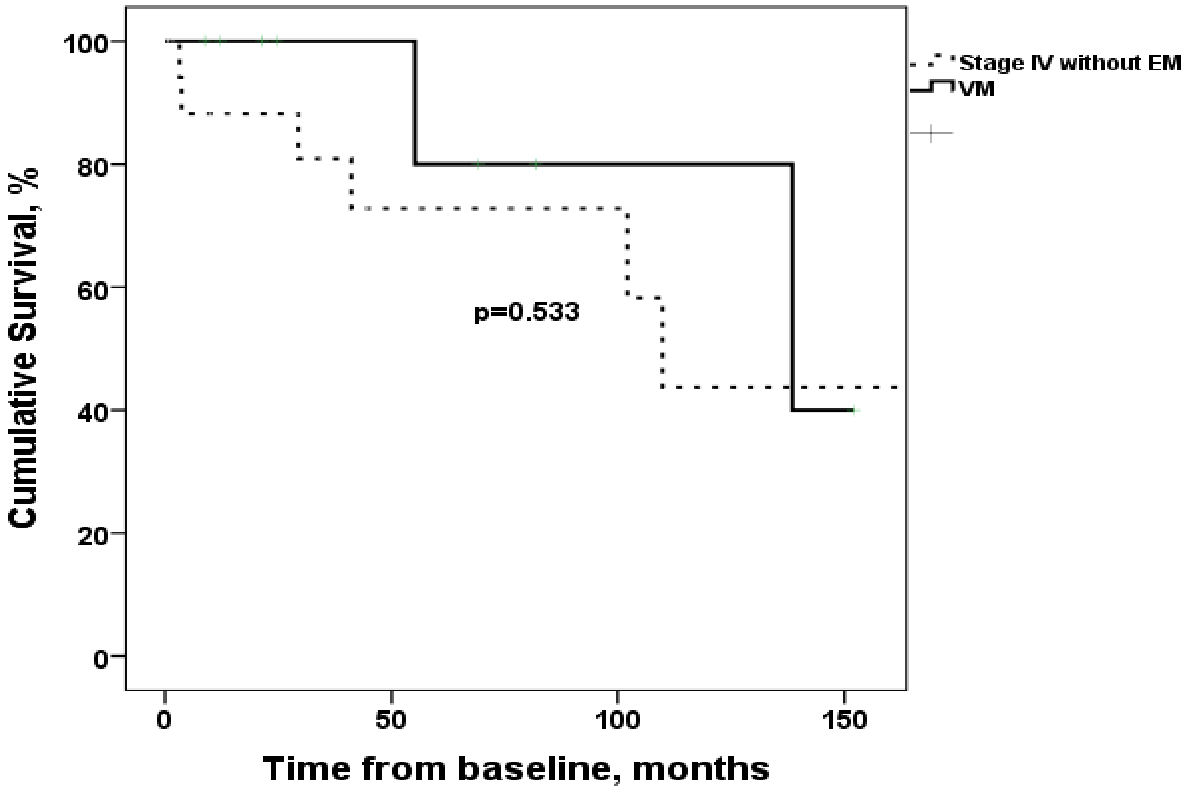

No. patients at risk.

**Virchow's node metastasis**	**9**	**5**	**2**	**1**
**Stage IV without extrahepatic metastases**	**18**	**8**	**5**	**2**

Supplementary Figure S2. Kaplan–Meier overall survival analysis of patients with small intestinal neuroendocrine tumors and Virchow's node metastasis and those matched for age and gender but without extrahepatic metastases. *P* = 0.533 (log rank test).

Abbreviations: EM, extra-abdominal metastases; VM, Virchow's metastasis
